# Left ventricular remodeling leads to heart failure in mice with cardiac‐specific overexpression of VEGF‐B_167_: echocardiography and magnetic resonance imaging study

**DOI:** 10.14814/phy2.13096

**Published:** 2017-03-29

**Authors:** Line Lottonen‐Raikaslehto, Riina Rissanen, Erika Gurzeler, Mari Merentie, Jenni Huusko, Jurgen E. Schneider, Timo Liimatainen, Seppo Ylä‐Herttuala

**Affiliations:** ^1^ Department of Biotechnology and Molecular MedicineA. I. Virtanen Institute for Molecular SciencesFaculty of Health SciencesUniversity of Eastern FinlandKuopioFinland; ^2^ Radcliffe Department of MedicineDivision of Cardiovascular MedicineUniversity of OxfordUnited kingdom; ^3^ Clinical Imaging CenterKuopio University HospitalKuopioFinland; ^4^ Gene Therapy UnitKuopio University HospitalKuopioFinland; ^5^ Heart CenterKuopio University HospitalKuopioFinland

**Keywords:** Cardiovascular imaging, heart failure, left ventricular hypertrophy, mouse ECG, vascular endothelial growth factor ‐B

## Abstract

Cardiac‐specific overexpression of vascular endothelial growth factor (VEGF)‐B_167_ is known to induce left ventricular hypertrophy due to altered lipid metabolism, in which ceramides accumulate to the heart and cause mitochondrial damage. The aim of this study was to evaluate and compare different imaging methods to find the most sensitive way to diagnose at early stage the progressive left ventricular remodeling leading to heart failure. Echocardiography and cardiovascular magnetic resonance imaging were compared for imaging the hearts of transgenic mice with cardiac‐specific overexpression of VEGF‐B_167_ and wild‐type mice from 5 to 14 months of age at several time points. Disease progression was verified by molecular biology methods and histology. We showed that left ventricular remodeling is already ongoing at the age of 5 months in transgenic mice leading to heart failure by the age of 14 months. Measurements from echocardiography and cardiovascular magnetic resonance imaging revealed similar changes in cardiac structure and function in the transgenic mice. Changes in histology, gene expressions, and electrocardiography supported the progression of left ventricular hypertrophy. Longitudinal relaxation time in rotating frame (T_1*ρ*_) in cardiovascular magnetic resonance imaging could be suitable for detecting severe fibrosis in the heart. We conclude that cardiac‐specific overexpression of VEGF‐B_167_ leads to left ventricular remodeling at early age and is a suitable model to study heart failure development with different imaging methods.

## Introduction

Death rates from cardiovascular diseases have declined, but the burden of disease remains high despite preventive actions and improved cardiovascular procedures (Go et al. 2014). Aging population and improved survival from other cardiovascular diseases increase the prevalence of heart failure (HF), which has transformed into a chronic disease often with a poor prognosis (Mudd and Kass 2008; Farmakis et al. 2015).

There is a clear need to develop imaging techniques for early diagnostics. Echocardiography has been the golden standard for evaluating left ventricular (LV) function, but the observer dependency and limitations in acoustic access and heart structure visualization impair the accuracy and reproducibility of the method (Jensen 2007). Three‐dimensional (3‐D) visualization of the LV by cardiovascular magnetic resonance imaging (CMR) has become useful in patients, where it is technically difficult to perform echocardiography and/or LV size and function are abnormal (Bellenger et al. 2000; Amundsen et al. 2011).

T_2_‐weighted CMR has been used to show the myocardial edema in acute infarction (Abdel‐Aty et al. 2004) and active inflammation in the myocardium (Mirakhur et al. 2013). Decrease in T_2_ value has not only been associated with interstitial collagen accumulation in the myocardium of diabetic mice (Loganathan et al. 2006) but also increased T_2_ value has been reported in spontaneously hypertensive rats with increased collagen deposition (Grover‐McKay et al. 1991; Caudron et al. 2013). Tissue characterization with late gadolinium enhancement (LGE) is often restricted due to contraindications to gadolinium. T_1_ relaxation in the rotating frame of reference (T_1*ρ*_) is a sensitive marker for macromolecular‐water interaction in environment with collagen and proteoglygan accumulation without the need for exogenous contrast agents (Witschey et al. 2012). However, very few studies reporting the use of T_1*ρ*_ in cardiac diseases have been published. Significant increase in T_1*ρ*_ time at the infarction site has been reported 1, 3, and 8 weeks after the acute incident (Witschey et al. 2012; Musthafa et al. 2013). A recent study applying T_1*ρ*_‐weighted imaging in patients suffering from hypertrophic cardiomyopathy revealed that the fibrotic area size measured from T_1*ρ*_ map correlated significantly with LGE‐positive areas (Wang et al. 2015). Fibrosis in noninfarcted myocardium is associated with pathological remodeling of the heart and the amount of fibrosis has been shown to correlate with hospitalization for HF, death, or both. Identification of the myocardial fibrosis would enable the use of therapies, which could reduce fibrosis and prevent adverse outcomes. (Diez et al. 2002; Schelbert et al. 2015.)

The role of vascular endothelial growth factor (VEGF)‐B in metabolism and cardiac function has been under investigation in recent years. Unlike transgenic rats expressing the human VEGF‐B gene or VEGF‐B_186_ isoform, cardiac‐specific overexpression of VEGF‐B_167_ in mice leads to the accumulation of ceramides in the heart (Karpanen et al. 2008; Kivela et al. 2014). The transgenic (TG) mice show concentric LV hypertrophy without compromising the systolic function of the heart until 1 year of age (Karpanen et al. 2008).

In this study, we showed that structural and functional changes in progressive LV hypertrophy can be detected by echocardiography and CMR. Electrocardiography showed depolarization and repolarization abnormalities connected to structural changes in LV. Elevated natriuretic peptide levels showed initiation of LV remodeling long before clinical signs of deteriorated systolic function. A correlation between diffuse fibrosis grading and T_1*ρ*_ relaxation time from CMR was found, and to best of our knowledge, this is one of the first studies using T_1*ρ*_ in imaging pathological remodeling of the heart.

## Materials and Methods

### Experimental animals

Five to 14 months old TG male mice with cardiac‐specific overexpression of human VEGF‐B_167_ on a C57Bl/6JOlaHsd background (*n* = 16) and their wild‐type male littermates (*n* = 15) were used to study the heart failure development with echocardiography and CMR. Transgene was targeted to the heart by a myocardium‐specific *α*MHC‐promoter (Karpanen et al. 2008). Eleven mice were imaged at all timepoints (5, 7, 10, 12, and 14 months) and killed at the age of 14 months. An additional 20 mice in total were imaged and killed either at 5 or 10 months of age to collect histological samples. The animals were kept in standard housing conditions in The National Laboratory Animal Center of The University of Eastern Finland. Diet and water were provided ad libitum. All animal experiments were approved by the National Animal Experiment Board of Finland and carried out in accordance with the guidelines of the Finnish Act on Animal Experimentation. The animal experiments conformed with *the Guide for the Care and Use of Laboratory Animals* published by the US National Institutes of Health (NIH Publication No. 85‐23, revised 1996).

### Echocardiography and electrocardiogram

Echocardiographic measurements were performed at the age of 5, 7, 10, 12, and 14 months using a Vevo770 Ultrasound System (VisualSonics Inc., Toronto, ON, Canada) and the electrocardiogram (ECG, lead II) was monitored during the echocardiography as described earlier (Huusko et al. 2010). Briefly a high‐frequency ultrasound probe (RMV‐707B) operating at 30 MHz, with a focal depth of 12.7 mm was used. The animals were anesthetized with isoflurane (induction: 4.5% isoflurane, 450 ml/min air, maintenance: 2.0% isoflurane, 200 ml/min air, Baxter International Inc., Deerfield, IL) during the imaging and ECG monitoring. Ejection fraction (EF), LV mass, LV diastolic wall thickness, and LV volume in diastole were determined from parasternal short‐axis (SAX) M‐mode measurements. EF was calculated by the Vevo770 software using the Teicholz formula.

Electrocardiogram (ECG) analyses were performed as described thoroughly in Merentie et al. (2015). Briefly, an ECG sample of 30 s of each mouse was analyzed and time intervals (PQ, QRSp, and QT; PQ represents the same as commonly used PR) were measured from the mean curve of the ECG sample with a specially made MatLab analysis program (The MathWorks Inc., MA; Kubios HRV analysis program version 2.0 beta 4, Department of Physics, University of Eastern Finland; Tarvainen et al. 2014; Merentie et al. 2015).

### Cardiovascular magnetic resonance imaging

All CMR experiments were performed at 9.4 T magnet, which was equipped with a Varian DirectDrive™ console (Varian Inc., Palo Alto, CA). Mice were placed prone on a pad filled with circulating warm water, and the pad inside the quadrature volume radiofrequency (RF) transceiver with coil diameter of 35 mm (Rapid Biomed, Rimpar, Germany). CMR experiments were performed at 5, 7, 10, 12, and 14 months of age.

The CMR protocol consisted of multi‐slice SAX gradient echo cine imaging, T_2_ mapping, and T_1*ρ*_ mapping. Cine images were acquired with gradient echo‐based sequence with repetition time 4.6 msec, echo time 2.1 msec, field‐of‐view 30 × 30 mm^2^, matrix size 256 × 256, and 1 mm slice thickness. Cine frames (15–18) were acquired within the ECG R‐R interval and 10 slices covered the whole myocardium. For T_1*ρ*_ adiabatic, a continuous wave sequence with nominal RF power of 29.3 mT (=1250 Hz), spin‐lock durations 0, 18, 36, and 54 msec were applied as previously described (Musthafa et al. 2013). The image readout was achieved by a segmented gradient echo sequence with repetition time 3.0 msec, echo time 1.6 msec, four k‐space lines were acquired after 1 sec delay and weighting pulse, field‐of‐view 30 × 30 mm^2^, and matrix size 128 × 128 points. T_2_ was measured similarly as T_1*ρ*_, but the spin‐lock pulse was replaced by adiabatic double spin echo sequence with echo times 0, 7, 14, 21, 28, and 35 msec. T_1*ρ*_ and T_2_ maps were created from 1‐mm thick SAX slice at the middle of the left ventricle.

The data were analyzed as described earlier (Musthafa et al. 2013). Briefly, LV volumes and functional parameters were calculated based on the end‐systolic and end‐diastolic cine frames. The left ventricle contours in systole and diastole were drawn in each slice from apex to valve level of the heart and the volumes were calculated to obtain end‐diastolic and end‐systolic volumes (EDV and ESV) of the LV and myocardial wall volume. EF [(1‐ESV/EDV) × 100] and LV mass (myocardium wall volume in diastole x conversion coefficient 1.05 g/cm^3^) were calculated manually. T_1*ρ*_ and T_2_ relaxation maps were reconstructed from image signal intensities and pixel‐by‐pixel manner and relaxation times were determined from the selected region of interest using a MatLab‐based software (The MathWorks Inc., MA; Aedes software package, aedes.uef.fi).

### Immunohistochemistry

Heart samples were immersion fixed in 4% paraformaldehyde‐15% sucrose for 4 h and in 15% sucrose overnight. A five micrometer thick paraffin‐embedded sections were stained to analyze myocardial fibrosis (Masson Trichrome, Accustain trichrome stains, Sigma‐Aldrich), apoptosis (Cleaved caspase‐3, Asp175, dilution 1:250, Cell Signaling Technology), proliferation (Ki‐67, ab15580, dilution 1:200, Abcam, UK), and glycogen accumulation (Periodic acid–Schiff′s glycogen staining, Sigma‐Aldrich). The amount of fibrosis was evaluated by three independent observers from Masson Trichrome‐stained sections in a blinded fashion and graded on a scale 0–3 as follows: 0 =  no fibrosis, 1 =  minor fibrosis, 2 =  moderate fibrosis, and 3 =  severe fibrosis. The number of apoptotic cells was calculated from five microscopic fields of cleaved caspase‐3‐stained sections and proliferating cells of Ki‐67‐stained sections within each animal at ×400 magnification. Glycogen accumulation was quantified from three microscopic fields of glycogen‐stained sections within each animal at ×200 magnification. All microscopic pictures were taken from the site of maximum staining in each section. Photographs of the sections were taken with an Olympus AX70 microscope (Olympus, Tokyo, Japan) and Eclipse Ni‐E Nikon microscope (Nikon Instruments Inc., NY).

### Quantitative RT‐PCR

Total RNA was isolated from the apex of left ventricle with TRI‐Reagent (Sigma‐Aldrich, St Louis, MO). Quantitative RT‐PCR was performed on a StepOnePlus Real‐Time PCR system (Applied Biosystems, Carlsbad, CA). Relative mRNA expression of atrial natriuretic peptide (ANP), brain natriuretic peptide (BNP), cardiac troponin T (cTnT), and peptidylprolyl isomerase A (PPIA) were measured using specific Assays‐On‐Demand systems (Applied Biosystems, Carlsbad, CA). Expression levels were normalized to PPIA.

### Clinical chemistry

Sodium, potassium, lactate dehydrogenase (LDH), and cTnT were measured from plasma samples collected on the killing day at 5, 10, and 14 months of age (Movet Oy, Kuopio, Finland).

### Statistical analyses

Results are presented as mean ± SD. Data were analyzed using linear mixed model analysis (IBM Corp. Released 2010. IBM SPSS Statistics for Windows, Version 19.0. Armonk, NY: IBM Corp.) or Student′s t‐test. Correlations between fibrosis grading and T_1*ρ*_ relaxation time or fibrosis grading and T_2_ relaxation time were studied with the GraphPad Prism5 software by calculating the value of the Pearson correlation coefficient (r). The used analyzing method is stated in the figure legends. *P* < 0.05 was considered statistically significant. The following symbols are used in the figures: *P < 0.05, **P < 0.01, ***P < 0.001.

## Results

### Expression of natriuretic peptides increased in TG mice already at the age of 5 months

**Figure 1:**
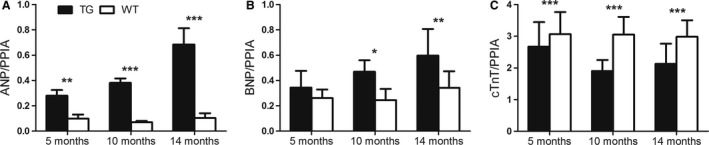
Relative mRNA expression of atrial natriuretic peptide (ANP), brain natriuretic peptide (BNP), and cTnT during left ventricular remodeling. Relative expression of (A) atrial natriuretic peptide (ANP) was increased at all ages and (B) brain natriuretic peptide (BNP) was increased at the age of 10 and 14 months in transgenic (TG) mice compared to wild‐type (WT) mice. (C) The relative expression of cardiac troponin T (cTnT) was decreased at all ages among TGs compared to WTs. mRNA expression of ANP, BNP, and cTnT were measured by RT‐PCR and normalized to peptidylprolyl isomerase A (PPIA). Mean ±  SD, statistical analyses with SPSS linear mixed model analysis. *P < 0.05, **P < 0.01, ***P < 0.001. TG *n* = 5–6, WT *n* = 5.

In TG mice, increased expression of ANP mRNA was detected at 5, 10, and 14 months of age (Fig. 1A) and increased expression of BNP at 10 and 14 months of age (Fig. 1B) when compared to WT group. cTnT was decreased in TG group at all timepoints as compared to WT group (Fig. 1C).

There were no differences between TG and WT mice in sodium and potassium levels in plasma at any timepoint (data not shown). LDH was significantly higher in TG group compared to WT at the age of 14 months (TG 746 U/l and WT 292 U/l, *P < 0.05, SPSS linear mixed model analysis), but at earlier timepoints, there were no differences (data not shown). Troponin T was measured from plasma but it was undetectable in both groups.

### LV volume and mass were increased and EF decreased in aged TG mice

**Figure 2:**
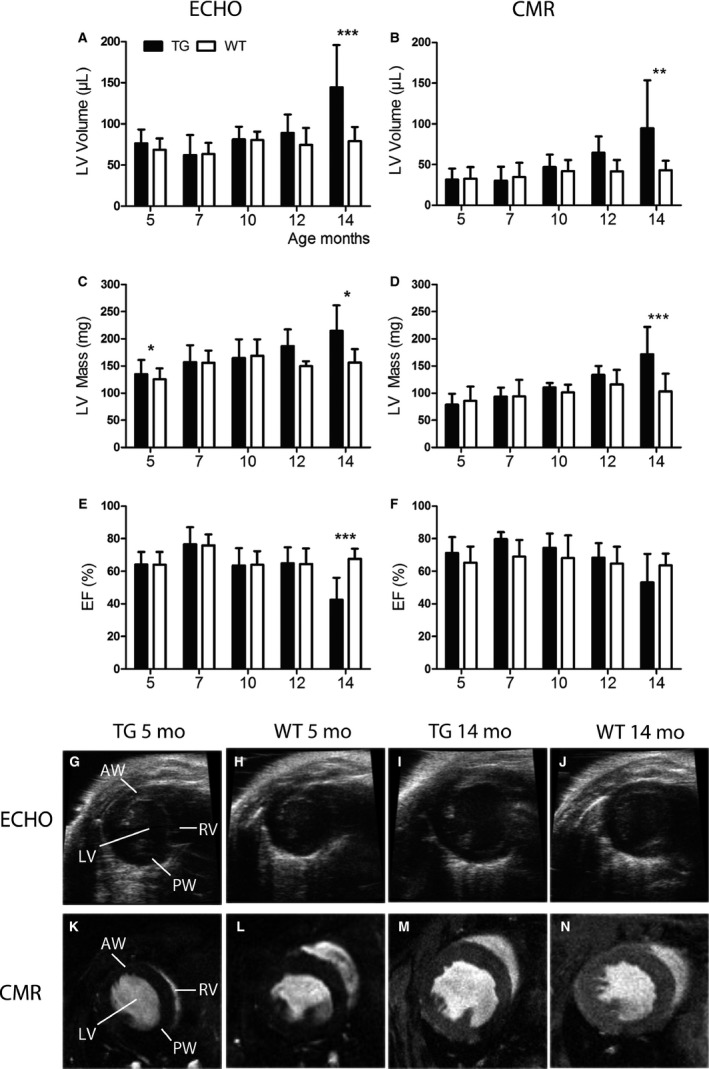
Measurements of cardiac structure and systolic function by echocardiography and cardiovascular magnetic resonance imaging (CMR). Left ventricular (LV) volume measured by (A) echocardiography and (B) CMR increased slowly with aging in transgenic (TG) mice and a marked dilatation of the LV was observed at the age of 14 months compared to wild‐type (WT) mice. (C, D) LV mass increased with aging in TG mice and at the age of 14 months, the difference compared to WTs was significant measured by both imaging methods. Ejection fraction (EF), measured with (E) echocardiography, decreased significantly in TG mice at the age of 14 months and the trend was similar when imaged with (F) CMR. Representative pictures from TG and WT mice imaged with (G–J) echocardiography and (K–N) CMR at the age of 5 and 14 months. The measurements were done from the mid‐ventricle level at end‐diastole. Mean ±  SD, statistical analyses with SPSS linear mixed model analysis. *P < 0.05, **P < 0.01, *** *P* < 0.001. TG *n* = 6–11, WT *n* = 5–10.

Heart structure and systolic function in TG mice overexpressing VEGF‐B_167_ in a cardiac‐specific manner and their WT littermates were observed by echocardiography and CMR in 5, 7, 10, 12, and 14 months of age (Fig. 2A‐N). LV volume, LV mass, and EF showed similar trends in results with both imaging methods.

LV volume (Fig. 2A, B) increased slowly in the TG group and a clear dilatation occurred between 12 and 14 months. LV volume remained unchanged in WT group at all timepoints compared to 5‐month baseline. Significant difference between the groups was seen at the age of 14 months, when LV volume measured by echocardiography was 144 ± 51 *μ*L in TG mice and 79 ± 17 *μ*L in WT mice compared to CMR measurements of 95 ± 59 *μ*L and 43 ± 11 *μ*L. Increase in LV volume in TG mice from 5 to 14 months of age was 89% (= 68 *μ*L, *P* < 0.001) measured by echocardiography and 199% (= 63 *μ*L, *P* < 0.001) measured by CMR. Similarly, LV mass increased slowly in TG group with aging (Fig. 2C, D) and was significantly different between TGs and WTs at 14‐month timepoint detected with echocardiography (TG 215 ± 47 mg and WT 156 ± 25 mg) and CMR (TG 172 ± 50 mg and WT 103 ± 32 mg). Percentage increase in LV mass in TG mice from 5 to 14 months of age was 59% (= 79 mg, *P* < 0.01) measured by echocardiography and 118% (= 93 mg, *P* < 0.001) measured by CMR. At the age of 14 months, significantly enlarged cardiomyocytes were detected in TG mice when compared to WT mice. On average, TG mice had 74 cardiomyocytes and WT mice had 93 cardiomyocytes in one microscopic view of 78.5 mm^3^ (*P* < 0.001 by Student′s t‐test, data not shown). LV anterior wall or posterior wall thickness did not differ significantly between TG and WT mice at any timepoint (data not shown).

Systolic function measured by EF (Fig. 2E, F) was significantly impaired in the TG group at the age of 14 months as measured by echocardiography (TG EF % 42 ± 14 and WT EF % 68 ± 6). However, EF did not differ between TG and WT mice at the earlier timepoints. The decreased EF was also seen with CMR at the 14‐month timepoint (TG EF % 53 ± 17 and WT EF % 63 ± 7), but the difference compared to WTs was smaller than seen in echocardiography. Decrease in EF in TG mice from 5 to 14 months of age was 34% measured by echocardiography (22 percentage points, *P* < 0.001) and 25% measured by CMR (18 percentage points, *P* not significant).

### Aged TG mice developed diffuse fibrosis in the heart

**Figure 3:**
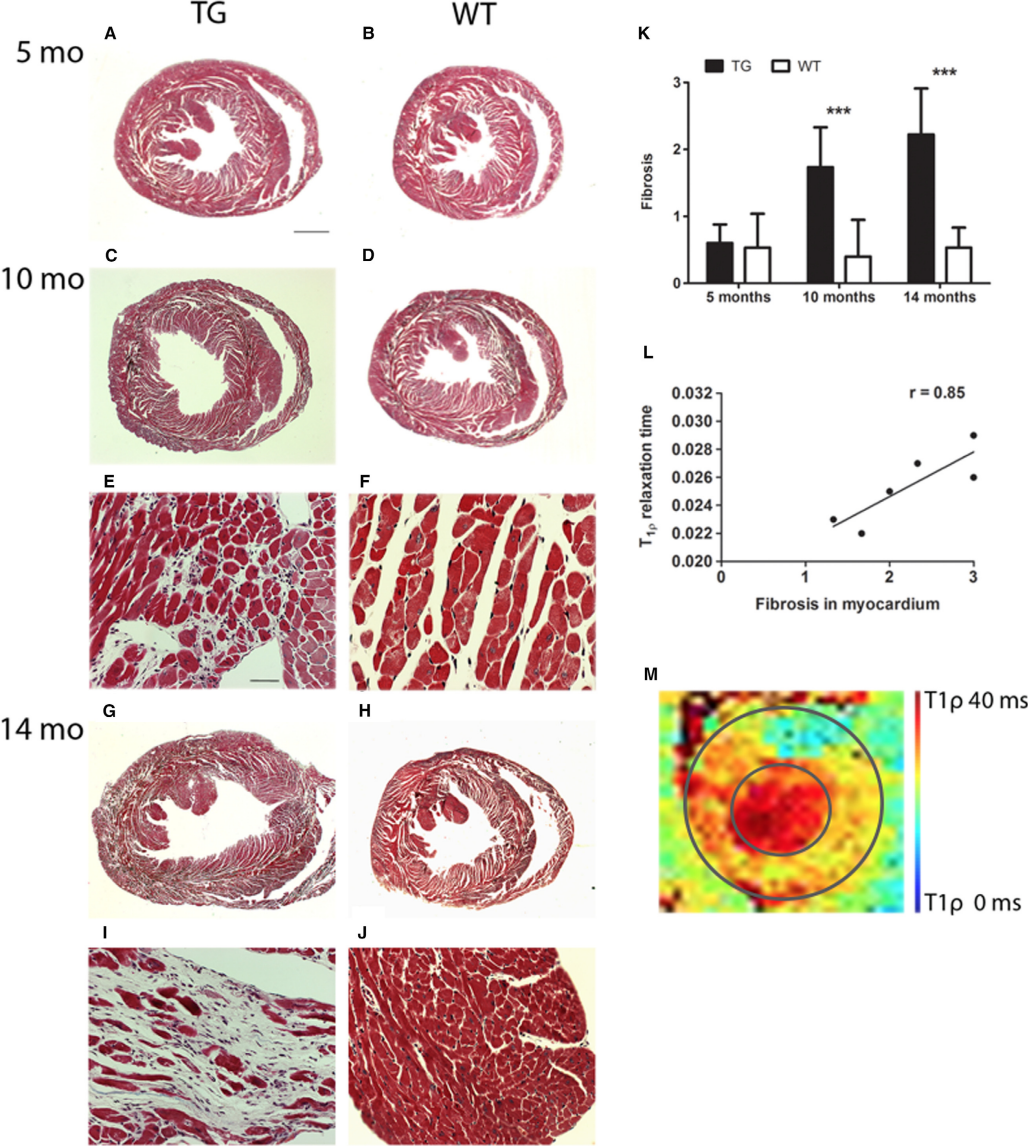
Progression of left ventricular hypertrophy led to myocardial fibrosis in transgenic (TG) mice. (A–K) Significant difference in the amount of diffuse myocardial fibrosis was detected from TG mice at the age of 10 and 14 months compared to wild‐type (WT) mice, which maintained normal morphology in all timepoints. (K) Fibrosis was graded from Masson trichrome‐stained sections by three independent observers on a scale 0–3, in which 0 =  no fibrosis, 1 =  mild fibrosis, 2 =  moderate fibrosis, and 3 =  severe fibrosis. (L) Fibrosis grading correlated with T_1*ρ*_ relaxation time measured from the left ventricle in TG mice by cardiovascular magnetic resonance imaging at the age of 14 months (Pearson r = 0.85, * *P* < 0.05). (M) Representative reconstructed T_1*ρ*_ relaxation time map from 14 months old TG mice; the average T_1*ρ*_ relaxation time was measured from the whole left ventricle. The inner circle represents endocardium and the outer circle epicardium. (A–D, G, H) Scale bar 1000 *μ*m and (E, F, I, J) 50 *μ*m. Mean ± SD, TG and WT mice were compared with Student′s t‐test at each timepoint. *** *P* < 0.001. TG *n* = 5–6, WT *n* = 5.

Progression of LVH led to diffuse myocardial fibrosis in TG mice, whereas WT mice maintained the normal morphology in the heart in all timepoints. Fibrosis was graded from Masson trichrome‐stained sections (Fig. 3A‐J) and a significant difference between TG and WT mice was detected at 10 and 14‐month timepoints (Fig. 3K). No significant differences in TG and WT mice were observed, when average relaxation times from reconstructed T_1*ρ*_ and T_2_ maps from CMR images were compared (data not shown). However, correlation (*r* = 0.85, *P* < 0.05) between the fibrosis grading and T_1*ρ*_ relaxation time in TG mice at the age of 14 months was found in this study (Fig. 3L, M). No correlation was observed between fibrosis grading and T_2_ time (data not shown).

Systolic function of the heart deteriorated at the age of 14 months, but no significant differences were observed in glycogen accumulation (evaluated from PAS‐stained sections), number of apoptotic cells (caspase‐3 staining), or number of proliferating cells (Ki‐67 staining) when TGs and WTs were compared (data not shown).

### LV fibrosis and dilatation in TG mice affected electrical function of the heart measured by electrocardiogram

**Figure 4:**
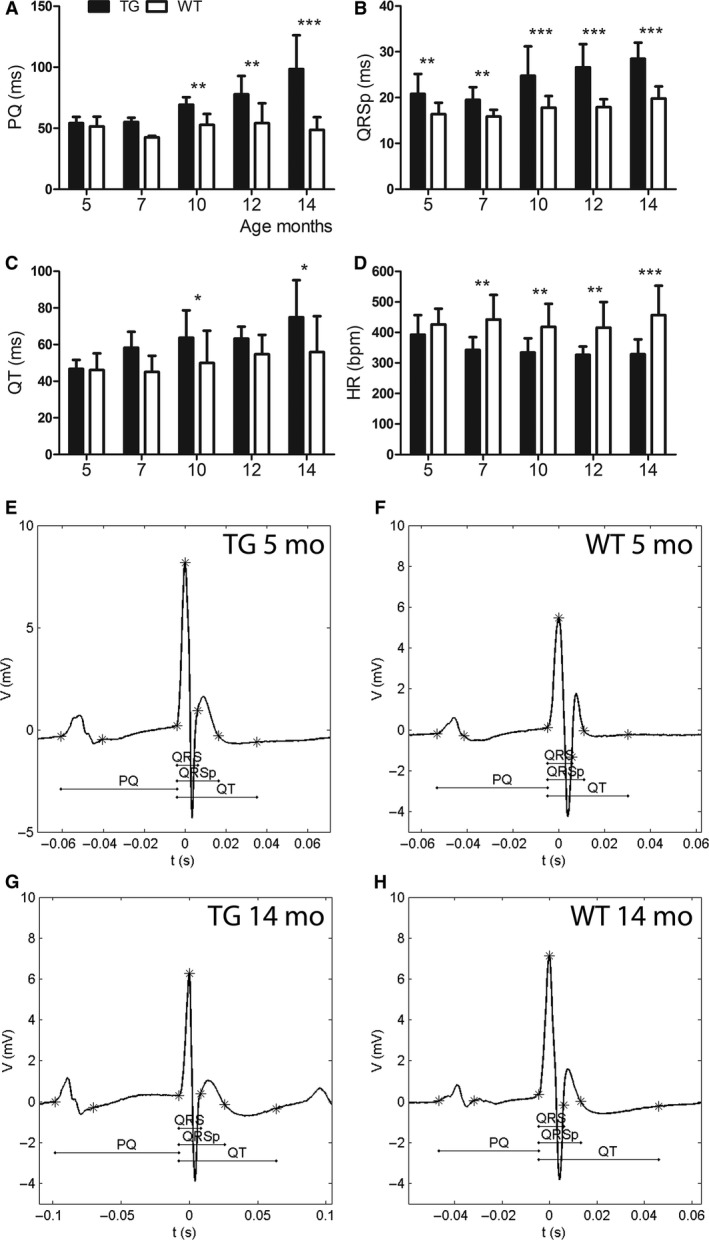
Changes in electrocardiogram (ECG) during left ventricular (LV) remodeling. (A) PQ interval started to prolong after 7 months of age and (B) QRSp time was significantly longer at all ages in transgenic (TG) mice when compared to wild‐type (WT) mice. (C) TG mice had longer QT interval at the age of 10 and 14 months and (D) lower heart rate than WT mice after 5 months of age. Representative ECGs from TGs and WTs at the age of (E, F) 5 months and (G, H) 14 months. Mean ± SD, statistical analyses with SPSS linear mixed model analysis. * *P* < 0.05, ** *P* < 0.01, *** *P* < 0.001. TG *n* = 6–11, WT *n* = 5–10.

PQ interval, similar to PR interval which shows the time from the beginning of atrial depolarization to the beginning of ventricular depolarization, started to prolong after 5 months of age and difference compared to WT mice was significant at 10, 12, and 14 months of age (Fig. 4A, E–H). TGs had significantly longer QRS time at the age of 14 months indicating changes in ventricular depolarization (data not shown). QRSp time referring to ventricular depolarization and early repolarization was significantly longer in TG mice compared to WT mice at all ages showing that repolarization was affected earlier than depolarization (Fig. 4B, E–H).

QT interval, representing the duration of ventricular depolarization and repolarization tended to prolong in TG mice with aging (Fig. 4C, E, G). The corrected QT interval QTc [mean QT/(RR/100)^1/2^] (Mitchell, Jeron & Koren 1998) showed the same trend at all timepoints as the absolute value of QT, which is preferred in anesthetized mice (Speerschneider and Thomsen 2013). TG mice had significantly lower heart rate after 5 months of age compared to WT mice (Fig. 4D).

In TG mice, the prevalence of premature atrial complexes (PACs) was 9% in 10 months old, 33% in 12 months old, and 17% in 14 months old mice. One out of six TG mice developed second‐degree atrioventricular (AV) block at the age of 12 months and at the later timepoint, it proceeded to third‐degree AV block. In WT mice, no PACs were detected, however, a single premature ventricular complex (PVC) was seen in one 14 months old WT mouse during ECG registration. Third‐degree AV block was also detected from one WT mouse at the age of 10 months.

## Discussion

HF is a chronic disease with a poor prognosis and there is a clear need to develop imaging techniques for early diagnostics. Therefore, we compared echocardiography and different CMR methods in detecting the progression of LVH leading to HF in mice with cardiac‐specific overexpression of VEGF‐B_167_. T_1*ρ*_ CMR was studied in detecting diffuse myocardial fibrosis. Results from ECG and gene expression analyses were combined to findings from the imaging data.

### Remodeling of the left ventricle in mice with VEGF‐B_167_ overexpression

Natriuretic peptides ANP and BNP, expressed in response to increased wall stretch in the heart and factors regulating hypertrophy, are known to reduce the peripheral resistance and volume load by increasing vasorelaxation, natriuresis, diuresis, and lowering blood pressure (Tavi et al. 2001; Kerkela et al. 2015). The systemic effects of ANP and BNP were shown as lower blood pressure (Karpanen et al. 2008) and heart rate in the studied TG mice compared to WT mice. Detected increase in the ANP and BNP expression has been connected to the remodeling process of the LV leading to cardiac hypertrophy and fibrosis (Kerkela et al. 2015), which could indicate that in TG mice, remodeling was ongoing already at the age of 5 months. Pathological remodeling seems to be associated with long‐term overexpression of VEGF‐B_167_ isoform in the heart, because LVH induced by overexpression of human VEGF‐B gene or VEGF‐B_186_ in rat heart does not affect cardiac function or expression of hypertrophic markers even at older age (Kivela et al. 2014). Overexpression of VEGF‐B_167_ isoform has shown to correlate with advanced pathological stage measured by tumor growth and invasiveness in hepatocellular carcinoma (Kanda et al. 2008) indicating also that the adverse effects of VEGF‐B_167_ transgene are connected with the level of the overexpression.

Interestingly, in this study, TG mice had significantly lower mRNA levels of cTnT already from the age of 5 months, which could indicate contractility defects. cTnT, the subunit which connects the troponin complex to tropomyosin, is known to regulate the calcium‐mediated interaction between actin and myosin and has a crucial role in regulating the contractility of the heart (Zhang et al. 2011). Several mutations in the gene coding cTnT are known to cause familial hypertrophic cardiomyopathy or dilated hypertrophy (Gomes et al. 2004). TG mice having lower cTnT expression in the heart have been shown to have increased ANP transcription, interstitial fibrosis, and diastolic dysfunction. Mitochondrial dysfunction due to degeneration and lipid accumulation were also observed in mice with reduced levels of cTnT. (Tardiff et al. 1999). Increased ANP expression and diffuse fibrosis shown in this study and formerly reported mitochondrial dysfunction (Karpanen et al. 2008) in mice with cardiac‐specific overexpression of VEGF‐B_167_ could be associated with the decrease in cTnT expression.

### Imaging the progression of left ventricular hypertrophy

Technical difficulties in performing echocardiography associated with abnormal LV structure and function have made 3‐D visualization of the LV by CMR a favored method for patients with cardiac disease (Bellenger et al. 2000; Amundsen et al. 2011). In our study, both echocardiography and CMR showed the LV mass increase in hypertrophy and LV dilatation attached to HF progression, however, the percentual increases in LV mass and volume from 5 to 14 months of age in TG mice were higher when measured by CMR. Contrary to previously reported concentric LVH in mice with cardiac‐specific overexpression of VEGF‐B_167_ (Karpanen et al. 2008), we did not detect differences in LV anterior or posterior wall thicknesses between TG and WT mice at any timepoint, which indicates eccentric type of LVH with increased LV volume in this study. TG mice were originally in FVB background and our TG mice were further bred into C57Bl/6JOlaHsd background, which seems to be the differing factor between these studies. Background of FVB or C57Bl has been shown to have influence on the phenotype in other TG mice in previous publications (Rose‐Hellekant et al. 2002; Haluzik et al. 2004), and this may explain by an unknown mechanism the different LVH type detected in this study.

Decrease in EF at the age of 14 months in TG mice was detectable with both imaging methods, but echocardiography gave lower values than CMR, which has been shown earlier in infarcted heart (Bellenger et al. 2000). 3‐D echocardiography has been shown to be more accurate and reproducible in measuring LV volumes and EF than two‐dimensional (2‐D) echocardiography, which is an often used method in adult cardiology (Dorosz et al. 2012). In human studies, echocardiography has shown smaller end‐diastolic and end‐systolic volumes but higher EF values compared to CMR (Wood et al. 2014). Contrary to humans, in the rat heart, EF was lower with 2‐D echocardiography than with CMR and the difference increased when infarcted heart was imaged (Stuckey et al. 2008). In small‐animal imaging, M‐mode echocardiography in mice showed thicker anterior and posterior walls in diastole indicating an increased mass when compared to CMR (Amundsen et al. 2011). The variability in the measured values from echocardiography and CMR is thus known from the previous studies.

The analysis method differs between echocardiography and CMR, which may partly explain the differences in results. Echocardiographic measurements are more prone to errors, as the slice selection for one‐dimensional M‐mode measurements during imaging has a major impact on the outcome. In CMR analysis, the whole LV can be analyzed objectively with making less assumptions about the heart structure as with echocardiography (Schneider et al. 2006). In diseased heart, CMR would be a preferable imaging method, although echocardiography due to its easy availability can be used for screening mice or patients in the clinic for further imaging.

### Detection of fibrosis with cardiovascular magnetic resonance imaging

Fibrosis is associated with the pathological remodeling of LV and HF progression. Early detection of fibrosis would be beneficial in diagnosing heart diseases, following the disease progression and enabling therapies, which could reduce the fibrosis in the heart (Diez et al. 2002). Indeed, diffuse fibrosis detected in the dilated LVs of TG mice at the age of 14 months in this study may explain the function loss in the heart.

T_1_ relaxation in the rotating frame of reference (T_1*ρ*_) is a sensitive marker for macromolecular‐water interaction in extracellular space with collagen and proteoglycan accumulation. High contrast with T_1*ρ*_ has been shown to be similar to LGE between myocardial infarction and remote myocardium, thus providing an imaging method for patients with contraindications for exogenous contrast agents. (Witschey et al. 2012; Musthafa et al. 2013). A higher signal to noise ratio in human hearts compared to mouse hearts results as better quality of the images in theory, however, low main magnetic fields typically used for human experiments may also induce different contrast between infarcted and remote myocardium. Low spatial resolution or long imaging time are the disadvantages in T_1*ρ*_ CMR compared to typical LGE imaging (Muthupillai et al. 2004). T_1*ρ*_ CMR in cardiovascular diseases has not been widely studied. In hypertrophic cardiomyopathy, large fibrotic areas detected with LGE CMR correlated with those measured by T_1*ρ*_ in patients (Wang et al. 2015), but in our study, diffuse fibrosis in TG mice did not change the average T_1*ρ*_ relaxation time significantly when compared to WTs. However, T_1*ρ*_ relaxation time from the whole myocardium tended to be increased like in fibrotic areas measured earlier (Witschey et al. 2012; Musthafa et al. 2013; Wang et al. 2015) and T_2_ relaxation time was unaltered in TG mice with significant diffuse myocardial fibrosis. Correlation between the severity of the diffuse fibrosis and T_1*ρ*_ relaxation time was shown in this study. This indicates that T_1*ρ*_ CMR might be useful in detecting moderate‐severe diffuse fibrosis from the whole myocardium.

### Changes in electrocardiography due to the progression of left ventricle hypertrophy

TG mice (14 months old) had widened QRS complex, indicating an increase in the duration of ventricular depolarization, which has been associated with LVH and HF in humans (Oikarinen et al. 2004) and mice (Boulaksil et al. 2010; Speerschneider and Thomsen 2013). QRSp duration, measuring LV depolarization and early repolarization, was prolonged at all timepoints in TG mice compared to WT mice indicating repolarization abnormalities already from 5 months of age in TG mice. Indeed, QRSp duration and LV mass have been shown to correlate in previous studies (Merentie et al. 2015). Prolonged QT interval in TG mice reflected partially the slow heart rate, but also LVH progression with aging. Time from the beginning of atrial depolarization to the beginning of ventricular depolarization (PQ interval) has been shown to be under 55–56 msec in healthy young mice (Kaese and Verheule 2012; Merentie et al. 2015). PQ interval >60 msec has been suggested to refer to the first‐degree AV block in mice (Merentie et al. 2015), which was detected from 10 months onwards in TG mice. It is known, that an aging or diseased heart is more prone to arrhythmias‐like first‐degree AV blocks, PACs, and PVCs shown in this study. In hypertrophic cardiomyopathy, supraventricular and ventricular arrhythmias have been shown to be frequent, but only complex ventricular arrhythmias had low positive predictive value for sudden death (Adabag et al. 2005). The electrocardiographic changes associated with cardiac‐specific overexpression of VEGF‐B_167_ have not been shown before to the best of our knowledge.

## Conclusion

We conclude that cardiac‐specific overexpression of VEGF‐B_167_ isoform in mice leads to HF. The associated changes to eccentric LV remodeling, namely an increase in LV mass and LV volume and a decrease in EF, could be visualized similarly with echocardiography and CMR. T_1*ρ*_ CMR showed a clear potential in detecting moderate‐severe diffuse myocardial fibrosis.

## Conflict of Interest

The authors declare no conflicts of interest.
